# Editorial: Computational Resources for Understanding Biomacromolecular Covalent Modifications

**DOI:** 10.3389/fcell.2021.728127

**Published:** 2021-07-14

**Authors:** Yu Xue, Hsien-Da Huang, Jiangning Song, Jian Ren, Dong Xu

**Affiliations:** ^1^Key Laboratory of Molecular Biophysics of Ministry of Education, Bioinformatics and Molecular Imaging Key Laboratory, College of Life Science and Technology, Huazhong University of Science and Technology, Wuhan, China; ^2^School of Life and Health Sciences, Warshel Institute for Computational Biology, The Chinese University of Hong Kong, Shenzhen, China; ^3^Biomedicine Discovery Institute and Department of Biochemistry and Molecular Biology, Monash University, Melbourne, VIC, Australia; ^4^School of Life Sciences, Sun Yat-sen University, Guangzhou, China; ^5^Department of Electrical Engineer and Computer Science and Christopher S. Bond Life Science Center, University of Missouri, Columbia, MO, United States

**Keywords:** biomacromolecular covalent modification, post-translational modification, DNA modification, RNA modification, machine learning, deep learning, data integration

Biomacromolecular covalent modifications (BCMs) include protein post-translational modifications (PTMs) and nucleic acid modifications. To date, 670 types of PTMs have been identified, and the most extensively studied PTMs are phosphorylation, ubiquitination, and acetylation. They are involved in regulating almost all biological processes, such as cell cycle, autophagy, and metabolism. More than 150 types of RNA modifications and tens of DNA modifications have been discovered, such as N^6^-methyladenosine (m^6^A) in messenger RNAs and 5-methylcytosine (5mC) in DNAs, and they play crucial roles in controlling gene expression. There is increasing evidence showing that PTMs are related to many diseases such as cancer and neurological disorders. RNA modification pathways are also found to be dysregulated in human cancer, and as such, epigenomic DNA modifications may shed some light on why certain diseases and tumors develop with aging.

The modification processes of diverse BMCs share some common properties. The deposition of chemical modifications (or marks) onto biomacromolecules is catalyzed by specific enzymes named “writers.” The enzymes that remove the modifications are called “erasers.” After recognizing the BCM sites, regulator proteins that produce a cellular response are “readers.” Identification of these BCM substrates and sites, as well as their “writers,” “erasers,” and “readers” can provide us a better understanding of how cellular activities are dynamically regulated. Computational algorithms, pipelines, tools, and databases play an increasingly important role in supporting biologists to explore BCM regulation, especially related regulatory mechanisms of protein PTMs and DNA/RNA modifications. We have witnessed substantial progress in computational development for BCM in both breadth and depth in recent years. The breadth covers a dramatically increasing number of BCM types, while the depth of new methods benefits extensively from the recent advancement in machine learning, especially deep learning. This Research Topic highlights these active developments with 8 predictive tools and 1 online database for BCMs in a timely manner. As shown in Figure 1, they represent a broad range of BCM types using various computational methods.

**Figure 1:**
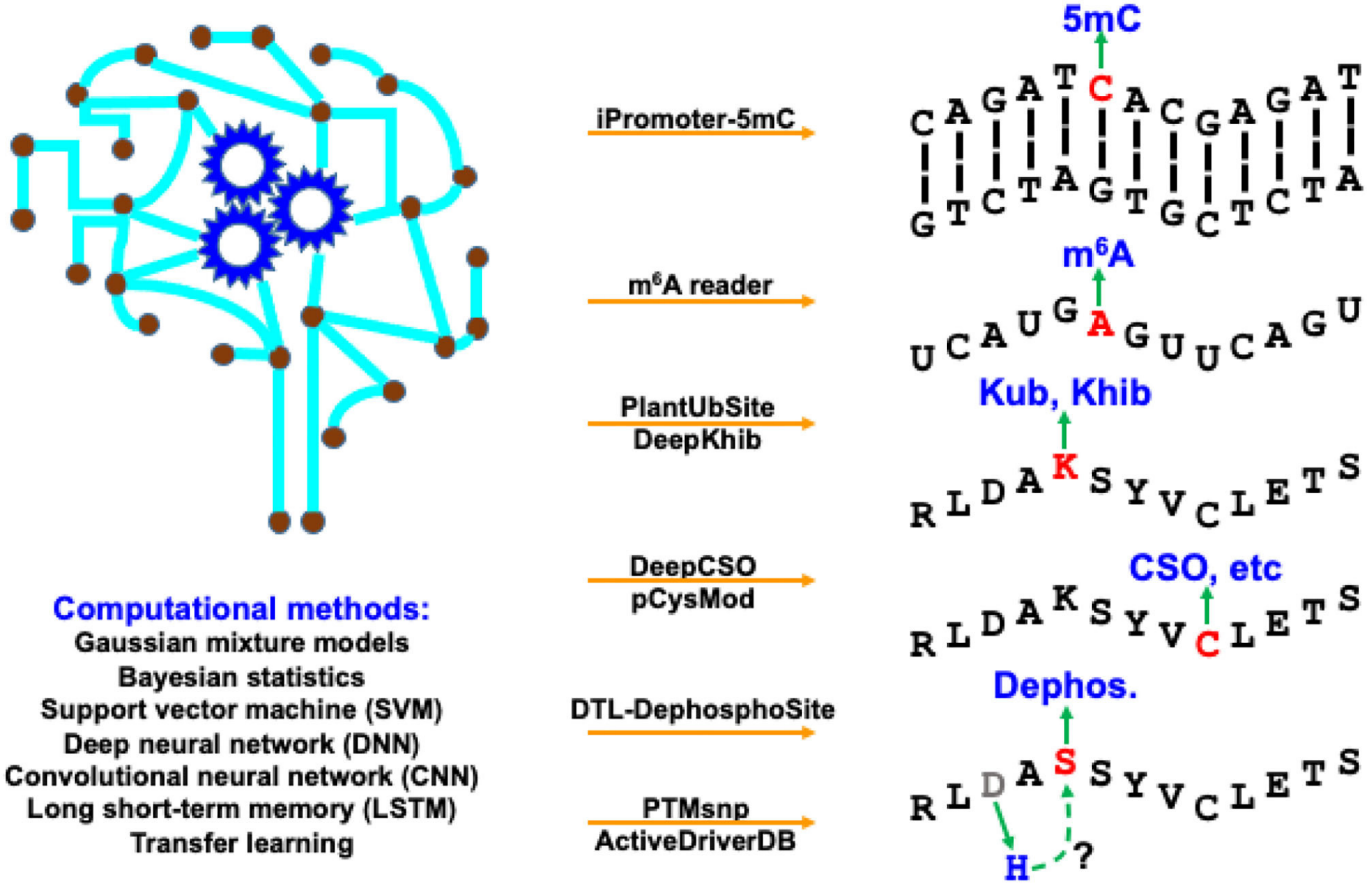
Computational resources covered in this Research Topic, as well as their targeting BCM types and applied methods.

In this Research Topic, two studies developed machine-learning frameworks to predict DNA/RNA modifications. iPromoter-5mC (https://github.com/zlwuxi/iPromoter-5mC) provides a deep neural network (DNN) framework to predict DNA 5-methylcytosine (5 mC) sites (Zhang et al.). The m6A reader (http://m6areader.rnamd.com) adopts a Support Vector Machine (SVM) model to predict reader-specific mRNA N^6^-methyladenosine (m^6^A) sites (Zhen et al.).

This Research Topic also presents five deep-learning tools for PTM predictions. Two studies target protein modification on the lysine (K) residue using convolutional neural network (CNN), including PlantUbSite (https://github.com/wang-hong-fei/DL-plantubsites-prediction) for predicting plant ubiquitylation sites (Wang et al.) and DeepKhib (http://www.bioinfogo.org/DeepKhib/) for predicting lysine 2-hydroxyisobutyrylation (Khib) sites (Zhang et al.). Two other studies target protein modification on the cysteine (C) residue: DeepCSO (http://www.bioinfogo.org/DeepCSO/) provides a long short-term memory (LSTM) tool to predict cysteine S-sulphenylation (CSO) sites in proteins (Lyu et al.), and pCysMod (http://pcysmod.omicsbio.info/) is a deep neural network (DNN) tool to predict multiple types of protein cysteine modification sites, including S-nitrosylation, S-palmitoylation, S-sulfenylation, S-sulfhydration, and S-sulfinylation (Li et al.). Furthermore, DTL-DephosphoSite (https://github.com/dukkakc/DTLDephos) provides an LSTM framework to predict dephosphorylation sites in proteins (Chaudhari et al.). All these studies demonstrate the excellent predictive power of deep learning for PTM predictions.

PTMsnp and ActiveDriverDB focus on functional annotations of the mutation effects on PTMs. PTMsnp (http://ptmsnp.renlab.org/) is an online service to predict driver mutations that potentially change PTM sites (Peng et al.). The authors use a Bayesian hierarchical model for the prediction, covering 411,574 sites from 33 types of PTMs and 1,776,848 somatic mutations. ActiveDriverDB (https://www.activedriverdb.org/) is an updated database of predicted PTM-specific impact of genetic variations based on Gaussian mixture models and Bayesian posterior probability estimation for proteins and their interaction networks (Krassowski et al.). An interesting estimate of the study indicates the widespread impact of PTM, i.e., 16–21% of pathogenic disease mutations, somatic mutations in cancer genomes and germline variants in the human population potentially affect PTMs and their downstream biological activities.

This Research Topic showcases state-of-the-art computational studies of BCMs. From these excellent papers, it is evident that the field is highly active and more research still is needed. We hope that readers can formulate some good ideas for future development from the papers or utilize the resources for their biological investigations. Finally, we, as the guest editors of this Research Topic, would like to thank all the authors for their valuable contributions.

## Author Contributions

YX and DX wrote the first draft. HH, JS, and JR provided critical comments and editorial suggestions for revisions. All the authors agreed on the submitted version.

## Conflict of Interest

The authors declare that the research was conducted in the absence of any commercial or financial relationships that could be construed as a potential conflict of interest.

